# Civil Malpractice

**Published:** 1873-02

**Authors:** M. A. McClelland

**Affiliations:** Knoxville, Illinois


A MONTHLY RECORD OF

*Medicine, Surgery and the Collateral Sciences.*

EDITED BY J. ADAMS ALLEN, M.D., LL.D.; AND WALTER HAY, M.D.

---

VOL. XXX. — FEBRUARY, 1873. — NO. 2.

---

### Original Communications.

ARTICLE I.—*Civil Malpractice. A Report presented to the Military Tract Medical Society, at its Fifteenth Semi-Annual Meeting, Jan.* 14, 1873. By M. A. MCCLELLAND, M.D., Knoxville, Illinois.

"Men see clearly, like owls, in the night of their own notions, but in experience, as in day-light, they wink and are but half-sighted."—BACON.

"Deep science is desirable to the man of fortune,—useful science to the physician and surgeon."—SIR ASTLEY COOPER.

"Professional employment is not only recognized as a legitimate and substantial business of life, but it is regulated by fixed rules to insure due diligence and skill and its appropriate reward."—SMITH *v.* HILL, *13 Ark. R. 173.*

---

INTRODUCTION.

Appointed on the Committee on Surgery, at the last semi-annual meeting of the Military Tract Medical Society, to prepare a report on some surgical topic, I considered in what way I could best serve the Association, whether by reporting on some special branch of surgery, on surgical appliances, or upon some of the civil relations surgeons bear to the public. Believing that I should serve our mutual interests best by choosing the last, I selected Civil Mal-practice as the subject of my report. What it is, I have endeavored to show, by collating its definitions from both legal and medical authorities. That the definitions, derived from legal sources, might not lose anything from their completeness, I have transcribed the decisions of the courts in full. Definitions from medical authorities have been somewhat abridged; as those for whom the report is written will have no difficulty in consulting the various authorities cited, they will not, therefore, desire the medical aspect of the case so much *in extenso.*

Free use has been made of both legal and medical works, and credit accorded the same. Any failure in this respect is due to oversight, which has especially been guarded against.

To Messrs. CRAIG, HARVEY, SANFORD, and KRETZINGER, of the Knox Co. Bar, I am under obligations for the use of legal authorities. Mr. KRETZINGER, especially, rendered me valuable suggestions and advice. T. S. MCCLELLAND, Esq., of the Chicago Bar, assisted me materially by furnishing Supreme Court Reports of other States, while my friend, Dr. REECE, of Abingdon, Ill., placed at my command surgical authorities and medical journals not in my own library.

Careful proof-reading has, we believe, kept the transcripts of decisions of the courts free from error, and as these decisions are only accessible in very large law libraries, we trust their presentation here will be of value.

---

CIVIL MALPRACTICE.

Circulars from our Medical schools very generally announce a course of lectures on Medical Jurisprudence. The announcement implies the promise of lectures upon the subject. How often this promise is kept, the memory of every medical practitioner will answer. The writer hereof, during his preliminary course of study, heard but two lectures upon this branch of medical science.

The import of the first was, that physicians generally received intimation sometime before they were called upon to act as experts in courts of law, or to respond in damages, and they would therefore have time to prepare themselves. The substance of the second was, "Get your fee as soon as possible after the service is rendered, and your patients will be better satisfied as to the result of your treatment."

Exposed as we constantly are to suits that will "ruin not only our fortunes, but our reputations," we receive too little instruction from our professional teachers, as to what we shall do to protect ourselves in the practice of our profession when once embarked in it. If any one doubts the necessity for an acquaintance with the general principles of the laws bearing upon the practice of Medicine and Surgery, let him consult the case of *Alder* v. *Buckley*, 1 *Swan's* (*Tenn.*) *R.* 69, and the other cases cited in this Report, and have his doubts resolved.

State medicine is too extensive, including, as it does, "the application of the principles and practice of the different branches of medicine to the elucidation of doubtful questions in courts of justice," to be considered in a paper of suitable length to be submitted to this Society. I shall therefore limit myself to the principles involved in malpractice.

This term means "an improper discharge of professional duty, either through want of skill or through negligence." The term is not a restricted one, but of wide application. Thus, the improper performance of work, requiring skill and knowledge, in any profession or business, would be designated "malpractice," and laws bearing upon it in all professions would be the same. *Duncan* v. *Blundell*, 3 *Starkie R.* 6. It would seem, however, from certain legal decisions, that more is expected of physicians and surgeons than of any other class of persons. Why should this be? Various reasons might be given in answer. In respect to surgical cases that are investigated in our courts, how frequently we hear surgeons—experts—boasting of the cures they have effected in similar cases; no shortening, no deformity, in their experience; leading the courts, leading juries, leading every one within the sound of their voice, to suppose that perfect cures were and should be the rule, and imperfect cures the exception. How far such statements are borne out by facts, we all well know. We also well know, that surgeons, generally, do not report their bad cases of shortening and deformity through our medical journals, hence the paucity of such cases from this source. Again, remarkable cures frequently result without any aid from us, but should we have but just seen the case, we often accept the credit that should have been given to nature alone. By just such a course we have impliedly warranted the *perfect* recovery of our patients; arrogating to ourselves powers never given to man; and having so done, we are held to the fulfillment of the contract. *Jones on Bailment*, 99; 3 *Black. Com.* 165. Do not understand me to bring these as specific charges against any gentleman of this Society; the charges are general ones, and arise necessarily from the state of our general society, that permits any one to practice our high calling, whether he be fit or no. What course is right for us in the premises? Can we do aught to release our profession from this reproach? Most certainly. In the language of a former President of this Society, (Dr. Reece,) "we can, by being candid in regard to our own deficiencies, claiming no more than we can perform; no more knowledge than we possess; no more for our art than belongs to it." It should ever be borne in mind that, "every surgeon sets a broken limb as he writes his name, after a fashion of his own," and that good results have, and may be obtained by a variety of methods of treatment.

Malpractice may conveniently be divided into two kinds, viz.: 1. "Civil Malpractice," in which patients bring suits for damages which they have, or think they have, sustained. 2. "Criminal Malpractice," in which the People, or State, is made the plaintiff. We shall, in the present paper, limit ourselves to the consideration of the first division of the subject. Having seen what constitutes malpractice, viz., "want of skill and negligence," we shall endeavor, first, to determine, from the rulings and decisions of the courts in adjudicated cases, what the law requires of us as surgeons; and second, what is considered skill and care by our leading authorities in medicine.

In an action for malpractice, the plaintiff always alleges that the defendant was either ignorant, that is, unskillful, or that he was negligent, that is, careless, or that he was both; and, as we shall see, the surgeon has been refused permission on diverse occasions to give proof of his competency—often the issue that is of most importance to him; hence, some of such cases have been taken to the higher courts, where this right has been recognized, and the case sent back for a new trial. It is in the lower courts, and from juries whose prejudices are usually with the plaintiff, that we have most to fear. Many good men occupy seats in our courts, who wish and aim to do right, and either from want of knowledge as to what should be expected of surgeons, or misled by the testimony of witnesses calling themselves "doctors" in medicine, give very erroneous instructions to juries; as in the case of *Howard* v. *Grover*, 28 *Maine R.* 97, where the jury assessed damages at $2,000 against the surgeon, not for unskillfully removing the limb, nor for any neglect in its after-treatment, but because in trying to save as much of the limb as possible—a prime principle in surgery—he did *not* remove it a few inches higher up. Here the surgeon was held responsible for an error in judgment. Few of us could follow our profession borne down by such requirements. *McClallen* v. *Adams*, 19 *Pick.* 333. See also article on "Attorney and Client," *Livingston's Law Mag.*, *Aug.*, 1855.

The law on responsibility of surgeons is well laid down in *Hilliard's Law of Torts*, *vol.* 1, *p.* 253. As it covers pretty generally all the points, alleged in cases of malpractice, I will transcribe it in full. He says: "Under some circumstances, a physician or surgeon will be held very strictly answerable for the consequences of his professional action or neglect. Thus, it is held that where medicine is administered to a slave without the consent of his owner, the physician is responsible for all the evil consequences which result from his act. So an action lies against a surgeon for gross ignorance and want of skill, as well as for negligence and carelessness; though if the evidence be of negligence only, which was properly left to the jury, and negatived by them, the Court will not grant a new trial because the jury were directed that want of skill alone would not sustain the action. But, in general, a physician or surgeon is responsible only for *ordinary* or *reasonable* care and skill, and the exercise of his best judgment in matters of doubt; not for a want of the highest degree of skill. It is the duty of the patient to co-operate with his professional adviser, and to conform to the necessary prescriptions; but if he will not, or under the pressure of pain he cannot, he has no right to hold his surgeon responsible for his own neglect. The implied contract of a surgeon is not to cure, but to possess and employ, in the treatment of a case, such reasonable skill and diligence as are ordinarily exercised in his profession by thoroughly educated surgeons; and in judging of the degree of skill required, regard is had to the advanced state of the profession at the time. So the law requires of a *dentist* a reasonable degree of skill and care in his professional operations; and he will not be held answerable for injuries arising from his want of the highest attainments in his profession. So a physician is expected to practice according to his professed and avowed system, where there is no particular system established or favored by law, and no system prohibited. Hence, in an action for malpractice, evidence to prove that the defendant's treatment of the case was according to the *botanic* system of practice and medicine, which he professed and was known to follow, is admissible."

What constitutes "ordinary skill," and what is proof of it? The phrase is a difficult one to define. There is no standard of comparison by which the question can be governed. Each individual case must stand upon its own merits. Time and place must be taken into consideration. As much cannot be expected of the physician in remote localities, where he is cut off from opportunities of improvement, as from physicians living in communities where opportunity is afforded of seeing disease and accidents under more varied forms; nor from this latter class will as high a degree of attainments be exacted as from physicians connected with large hospitals, or who reside in large cities. If it were otherwise, we should find but few physicians, except in populous communities. *Braunburger* v. *Cleis*, *Am. Law Reg.*, 1864-5, 587. The least amount of skill, therefore, with which a fair proportion of the practitioners of a given locality are endowed, is taken as the criterion by which to judge the physician's ability or skill. 1 *Bouvier's Inst.*, § 1004–5. In proof of this degree of attainment, a diploma is the best evidence, but to be valid it must be proven that the college from which it emanated had corporate authority to grant degrees in medicine at the date of giving the degree, and if the college of another State, its act of incorporation must be offered as proof of its authority to grant such degree. *Ordronaux*, *Med. Jurisprud.* 26; *Hunter* v. *Blount*, 27 *Georgia*, 76; *Hill* v. *Bowdie*, 2 *Stewart & Porter*, 56. It must be borne in mind, also, that courts will take no notice of the different schools in medicine, the term "physician" being legally assumed by any one who chooses to announce himself as a practitioner of medicine. *Sutton* v. *Tracy*, 1 *Mich.* 243; *Reynolds* v. *Graves*, 3 *Wis.* 416. The law recognizes all systems as legitimate; at the same time it requires the physician to practice according to his professed and avowed system. A departure from the received canons of a given system will be taken as a want of ordinary skill. *Bowman* v. *Woods*, 1 *Iowa*, 441; *Patten* v. *Wiggin*, 51 *Maine*, 594.

The Supreme Court of New York has decided, (*Ex parte Paine*, 1 *Hill*, 665,) that, "whoever offers to practice either homœopathy or allopathy, as his patients may wish, is practically a quack in his profession." This is but a single phase of it. The American Medical Association, in defining the duties for the support of professional character, takes a more general, and consequently more just, view of the above question. Sections 3–4 declare it "to be derogatory to the dignity of the profession to resort to public advertisements, or private cards, or hand-bills, inviting the attention of individuals affected with particular diseases; publicly offering advice and medicine to the poor gratis, or promising radical cures; or to publish cases and operations in the daily prints, or to suffer such publications to be made; to invite laymen to be present at operations, to boast of cures and remedies; to adduce certificates of skill and success; or to perform any other similar acts. These are the ordinary practices of empirics, and are highly reprehensible in a regular physician.

"Equally derogatory to professional character is it, for a physician to hold a patent for any surgical instrument or medicine; or to dispense a secret *nostrum*, whether it be the composition or exclusive property of himself or of others. For, if such nostrum be of real efficacy, any concealment regarding it is inconsistent with beneficence and professional liberality, and if mystery alone gives it value and importance, such craft implies either disgraceful ignorance or fraudulent avarice. It is also reprehensible for physicians to give certificates attesting the efficacy of patent or secret medicines, or in any way to promote the use of them."

Any one violating the above provisions would be designated a "quack;" so, also those practitioners of the "Specific School," those transcendental pathologists, who consider as signs of the same disease, and as possessing equal value, the following symptoms: "Insatiable thirst; pallor of the face; scrofula; sweating of the head after sleep; burning in the palms of the hands; frequent attacks of suffocation; furuncles; vomiting of blood; hiccough after eating or drinking; cutting pain in the rectum while at stools; absence of venereal desire; unbridled lusts; somnolency during the day after eating; paroxysms of anger bordering on mental alienation; tears frequently at the slightest causes," etc., etc.; and who assure us in their therapeutics, that the administration of the 1,000,000,000,000,000,000,000th of a grain of carbonate of lime—common chalk—produces no less than a thousand and ninety symptoms, from which I select the following: On the fifth day, "itching on the border of the eyelids; thirteenth day, *in the evening*, on going out, unsteady gait; seventeenth day, ardent venereal desires, especially during a walk *before dinner;* twenty-first day, great heat at the extremity of the big toe; twenty-eighth day, itching at the anterior part of the glans penis, after urination," and so on, *ad nauseam.*

What else can we call them but "quacks,"—ignorant pretenders to knowledge they do not possess? Is this overstrained? Read the history of medicine. I must ask pardon for this digression. The law seems inclined to make definitions. Legitimate medicine demands the same right, especially in its own peculiar province.

### ADJUDICATED CASES.

The case of *Bowman* v. *Woods*, 1 *Iowa*, 441, is a fine illustration of what these self-styled reformers are doing in the profession of medicine, and as it also illustrates some important points in law, I quote it entire.

The opinion of the court was given by GREEN, J.

"The proceedings below were against Bowman for malpractice as a physician in a case of accouchment. Verdict for the plaintiff, and his damages assessed at $50. The bill of exceptions gives the substance of a Dr. Coffin's testimony, who, it appears, was called in as consulting physician about thirty-six hours after the delivery. At that time Dr. Coffin states that 'the afterbirth was not removed, and the patient was greatly prostrated by the severity of the labor and loss of blood; that she was also suffering from a distension of the bladder, which had not been evacuated since parturition. He gave it as his opinion that the *placenta*, and the distended state of the bladder, should have been removed at a much earlier period, and that such delay would be likely to produce puerperal fever. Several other physicians, as witnesses, concurred in Dr. C.'s view of the practice.'

"The defendant then offered to prove that he was a *botanic* physician, and that according to the botanic system of practice and medicine, it is considered *improper* to remove the placenta, and that it should be permitted to remain till expelled by efforts of nature. But the proof of these facts being objected to, was ruled out by the court. In this we think there is error. As yet there is no particular system of medicine established, or favored, by the laws of Iowa; and as no system is upheld, none is prohibited.

"The regular, the botanic, the homœopathic, the hydropathic, and other modes of treating disease, are alike unprohibited; and each receives more or less favor and patronage from the people.

"Though the regular system has been advancing as a science for centuries, aided by research and experience, by experience and skill, still the law regards it with no partiality or distinguishing favor; nor is it recognized as the exclusive standard or test by which the other systems are to be adjudged. The evidence of the experienced practitioner of either system, is equally admissible in giving opinions upon questions of medical skill. But in the question before us, the objection does not appear to be the disqualification or skill of the witnesses, but rather the facts which the defendant proposed proving by them. In these facts we can see nothing irrelevant or inadmissible; and as matter of defense to the jury, the defendant was entitled to the benefit of them. A person professing to follow one system of medical treatment cannot be expected by his employer to practice any other. While the regular physician is expected to follow the rules of the old school in the art of curing, the botanic physician must be equally expected to adhere to his adopted method. But on the part of every medical practitioner, the law implies an undertaking that he will use an ordinary degree of care and skill in medical operations, and he is unquestionably liable for gross carelessness or unskillfulness in the management of his patients; and still the person who employs a botanic practitioner has no right to expect the same kind of treatment or the same kind of medicine that a regular physician would administer. The law does not require a man to accomplish more than he undertakes, nor in a different manner from what he professes. Therefore, in this case, if the defendant below could show that he was employed as a botanic physician, and that he performed the accouchment with ordinary skill and care, in accordance with the system he professed to follow, we should regard it as a legal defense. It should show a full compliance with his profession and undertaking, and if injury resulted from it to the plaintiff, he could properly blame no one but himself. Story, in his work on Bailment, Sec. 435, says: 'But even where the particular business or employment requires skill, if the bailee is known not to possess it, or he does not exercise the particular art or employment to which it belongs, and he makes no pretension to skill in it; then if the bailor, with full notice, trusts him with the undertaking, the bailee is bound only for a reasonable exercise of the skill which he professes, or of the judgment which he can employ; and if any loss ensue from want of due skill, he is not chargeable. Thus,' (to put a case borrowed from the Mohammedan law,) 'if a person will knowingly employ a common matmaker to weave or embroider a fine carpet, he may impute the bad workmanship to his own folly. So if a man having a disease in his eyes, should employ a farrier to cure the disease, and he should lose his sight by the remedies prescribed in such cases for horses, he certainly would have no cause for complaint.'

"Judge Story then goes on to state, that in all such cases the employer ought properly to attribute the loss or injury to his own negligence and mismanagement. The case of the *Commonwealth* v. *Thompson*, 6 *Mass.* 134, exhibits a revolting case of malpractice in which lobelia was administered to such indiscriminate excess as to produce death. Still it was held, that if a medical pretender administers medicine to his patient with an honest intention and expectation of cure, but which causes death, the party prescribing cannot be adjudged guilty; and that 'there is no law which prohibits any man from prescribing for a sick person, with his consent, if he honestly intends to cure him by his prescription.'

"The people are free to select from the various classes of medical men, who are accountable to their employers for all injuries resulting from a want of ordinary diligence and skill in their respective systems of treating diseases. It is to be lamented, that so many of our citizens are disposed to trust health and life to novices and empirics, to new nostrums and methods of treatment. But these are evils which courts of justice possess no adequate power to remedy. Enlightened public opinion and judicious legislation, may do much to discountenance quackery and advance medical science.

"The only other error assigned in this case, which we deem it necessary to notice, is in relation to the admission of medical books as evidence. It appears that the defendant offered to introduce certain medical books, which witnesses had declared as standard works on botanic medicine, and from which they claimed to have derived much of their professional knowledge, but, on objection, the court excluded them. The authorities on this point are not uniform; but the district judge decided in conformity to the prevailing decisions of at least the English courts. In the case of *Collier* v. *Simpson*, 5 *C. & Payne's N. P. R.* 73, it was decided that medical books are not admissible in evidence, though professional witnesses may be asked the grounds of their judgment and opinion, which might in some degree be founded on these books as a part of their knowledge.

"Judge Abbot, in the trial of *Donal* for poisoning, refused an appeal to the works of Thenard, and said, 'We cannot take the fact from any publication, we cannot take the fact as related by any stranger.' But in the trial of *Spencer Cooper*, the court permitted medical authorities to be read, (Guy's Forensic Medicine, 11); and Dr. Beck, in his excellent work on Medical Jurisprudence, vol. 2, p. 666, states, that in this country an objection has never been made to the introduction of authority, or the observation of others, as testimony, by medical men. In this we think the author mistaken, for an appeal to medical authorities has been disallowed by some of the courts of this country; though physicians, when testifying, are permitted to refer to medical authors, and to quote their opinions from memory. Being permitted to refer to and quote authors, we can see no reason why they may not read the views and opinions of distinguished authors. The opinion of an author as contained in his works, we regard as better evidence than the mere statement of those opinions by a witness, who testifies as to his recollection of them from former reading. Is not the latter secondary to the former? On the whole we think it the safer rule to admit standard medical books as evidence of the author's opinions upon questions of medical skill or practice, involved in a trial. This rule appears to us the most accordant with well established principles of evidence." *Judgment reversed.*

The ruling above, so far as it relates to the admission of medical books in evidence, is eminently just. It is from our teachers, and from our medical authorities, that we form our opinions and draw our conclusions in a vast majority of cases. There are often cases in our courts, concerning which our personal experience does not enable us to form opinions, and we certainly should at least be permitted to show that *our opinions*, when not formed from *our experience*, are sustained by the *written experience* of eminent men in our profession. Professor Lee remarks, that, "Medical testimony when of *any* value, is but little else than a reference to *authority*."

The rules of law governing cases of malpractice in Illinois, are laid down in the cases of *Ritchey* v. *West*, 23 *Ill.* 385, and *Lowe* v. *McNevins*, 40 *Ill.* 209. The opinions of the court in these cases are here given in full.

The evidence in the court below, before the jury in the last mentioned case, was that the plaintiff's (McNevins') arm was broken in the elbow-joint. The defendant (Lowe) bound up the arm with a bandage, and said it was all right; told Mr. and Mrs. McNevins to wet it frequently with vinegar and wormwood. He then left, and did not return to see his patient for several days. When he called again, he examined the arm, and said it was doing well. Mrs. McNevins discovered that the bones were sticking up, and sent for the defendant. He came and examined the arm again, and still claimed it was all right; that the bone which stuck up was attached to the skin and would come all right; and that they must rub it with oil. He said, also, that the patient would outgrow the deformity in five or six days. Said he had cured a broken leg in New Boston, that Drs. Willits and Harrell could not cure; also told of the great cures he had performed in Wisconsin, where he had a large practice. The jury found for the plaintiff; damages, $700. *Appealed.*

*Lowe* v. *McNevins*, 40 *Ill.* 209. THOMPSON, J. Mr. Justice LAWRENCE delivered the opinion of the court.

"This was an action brought against the appellant for malpractice as a surgeon and physician. In the third and fourth instructions for the plaintiff, the court told the jury that the defendant, if he held himself out as a physician, was liable for whatever damage may have accrued to the plaintiff by reason of *any* want of care or skill on his part, whether he charged fees or not. This states the responsibility of the physician too strongly, as it requires the highest degree of care and skill, whereas only reasonable care and skill are necessary.

"As to the payment of fees, the instruction is unobjectionable. If a person holds himself out to the public as a physician, he must be held to ordinary care and skill in every case of which he assumes the charge, whether in the particular case he has received fees or not. But if he does not profess to be a physician, nor to practice as such, and is merely asked his advice as a friend, or neighbor, he does not incur any professional responsibility. The case of *Ritchey* v. *West*, 23 *Ill.* 385, is to be understood in this sense. The judgment must be reversed because the instructions required the highest degree of care and skill." *Judgment reversed.*

This case was finally settled for $200, pending a new trial.

The case referred to in the above decision, was a suit brought by defendant in error, against plaintiff in error, to recover for injuries arising from negligence and lack of skill of the plaintiff in error, in the practice of his profession of physician and surgeon. The case was tried in the Adams Circuit Court, SIBLEY, J., presiding.

*Ritchey* v. *West*, 23 *Ill.* 385. WALKER, J. "No question can arise on the correctness of the decisions of the court below, in admitting or rejecting evidence, in this case, as no exceptions were preserved in the record.

"The principle is plain and of uniform application, that when a person assumes the profession of physician and surgeon, he must in its exercise be held to employ a reasonable amount of care and skill. For anything short of that degree of skill in his practice, the law will hold him responsible for any injury which may result from its absence. While he is not required to possess the highest order of qualification to which some men attain, still he must possess and exercise that degree of skill which is ordinarily possessed by members of the profession. And whether the injury results from want of skill, or want of its application, he will, in either case, be equally liable. This, the law implies, whenever a retainer is shown; but when the services are rendered as a gratuity, gross negligence will alone create liability.

"This, then, presents the question, whether the evidence in the case establishes a want of ordinary skill or reasonable care, in the treatment of this case. The retainer having been proved, it is not material to inquire whether the case shows gross incompetency or neglect of duty. The concurring evidence of all the physicians shows that the splints and bandages were not properly applied. Had they extended below the wrist, the evidence seems to show that they would have confined the wrist to its proper place. It is probable that such a practice would have tended, notwithstanding the fracture, to have held the broken bone more nearly to its place until a union was formed, and thus have prevented, to some extent, if not altogether, the deformity and disability to use the hand. The physicians also agree that the splints employed were not of sufficient width, as well as too short, for the treatment of the fracture, even if they had been midway between the wrist and elbow, as he supposed. And from this evidence it would seem that there must have been a want of ordinary skill or great negligence in the treatment of the case, in not detecting the dislocation of the wrist joint. The physicians all agree that this portion of the injury could have been easily detected by ordinary care and skill, and the fact that it had been and was still dislocated, was afterwards detected by a person who did not profess surgery or skill in such matters, and had previously only had slight experience in cases of fractured limbs. Then if the evidence of the medical men who were examined as witnesses, is to be credited, and it is supported by the fact that the dislocation of the wrist was detected by a person professing to have no skill, there was a want of ordinary care, or skill, or both, manifested in the treatment of the case.

"The medical witnesses all testify that it is customary and necessary for the surgeon to pay a second visit, for the purpose of ascertaining how the case is progressing, and whether further treatment is required, unless it be dispensed with by the patient. There was no conflict in the evidence, that the plaintiff in error was requested to return, which he agreed to do, for the purpose of further examination, on the following day, and that he never afterwards returned. Then the fact is established by the evidence, that he not only promised to return, but that it was his duty to have done so, unless notified that such attendance would be dispensed with. Then if this was a part of his professional duty, its omission establishes a want of reasonable care and diligence, which, together with his failing to comply with his agreement to return, must render him liable for all injury which has resulted from its non-observance. Had he returned, as his duty and his agreement required, in all probability the visit would have resulted in detecting the true situation of the injury, and relief might then have been obtained by the employment of the necessary surgical aid. The court therefore did right in refusing the twelfth instruction asked for by plaintiff in error, as it assumed that it was not his duty to again visit defendant in error.

"It is likewise urged, that the court below erred in refusing to grant a new trial on the affidavit of newly discovered evidence. The facts alleged in the affidavit to have been newly discovered, were only cumulative. The question tried by the jury was, whether the wrist was fractured and dislocated at the time when the plaintiff in error was called to treat the injury. The theory of the defense was, that the wrist was not then injured. This evidence which is said to be newly discovered, if it had been produced, would have only tended to show that the wrist received no injury at the time he was called for medical advice. The evidence of the witnesses of defendant in error was, that the wrist was then injured, and from which it had never recovered, and the newly discovered evidence was only rebutting, and was cumulative to his other evidence of that character.

"But if this were not true, the affidavit was fatally defective in not stating that the evidence, said to be newly discovered, was true. If he was unable himself to swear to its truth, he should have produced the affidavits of the witnesses themselves, to satisfy the court of such truth. In an application for a new trial because of newly discovered evidence, it is not sufficient for the party to state that he has been informed and believes that the witnesses will testify to the facts, but the truth of such facts must be verified by affidavit. Otherwise but few cases would occur, in which a party might not procure some person to state that he would on the trial swear to the necessary facts to procure the new trial, and yet, when placed on the stand, wholly fail to testify in accordance with such statement. Such a practice would be liable to great abuse, and should not therefore be adopted.

"Upon the whole of this record we are unable to perceive any error for which the judgment of the court below should be reversed, wherefore the same is affirmed."

The law regarding what is required of the plaintiff in a suit against a physician for malpractice, is laid down in the case of *Patten* v. *Wiggin*, 51 *Maine*, 594, as follows:—opinion of the court containing, also, a clear statement of what the law requires of surgeons.

*Action*, *Assumpsit* on account annexed. One portion of account is for professional services as a physician, in attendance on defendant's minor son. The defense to this portion of the claim was malpractice in the treatment of the patient, and such ignorance, want of skill and judgment on the part of the plaintiff in managing professionally the case under his care, that the patient was more injured than benefited by his treatment, and that on the whole case he was not reasonably entitled to recover anything for his services.

Evidence was introduced on both sides as to such treatment and management by the plaintiff during the whole time the patient was under his care. The court (Judge KENT) instructed the jury that, if the plaintiff had been guilty of malpractice, or neglect, or want of ordinary care and skill, within the rules hereafter stated, it would be a defense to that part of the claim which related to the treatment of defendant's son, and the court instructed the jury as follows:

"1. When a man offers himself to the public, or to patients, as a physician or surgeon, the law requires that he be possessed of that reasonable degree of learning, skill, and experience, which is ordinarily possessed by others of his profession who are in good standing as to qualification, and which reasonably qualifies him to undertake the care of patients."

This rule does not require that he should have the highest skill, or largest experience, or most thorough education, equal to the most eminent of the profession in the whole country; but it does require that he should not, when uneducated, ignorant and unfitted, palm himself off as a professional man, well qualified, and go on blindly and recklessly to administer medicines, or perform surgical operations. The rule above stated is the true one.

But the physician qualified within this rule, may be guilty of negligence or malpractice.

"2. The law requires and implies, as part of the contract, that when a physician undertakes professional charge of a patient, he will use reasonable and ordinary care and diligence in the treatment of the case.

"3. The law further implies, that he agrees to use his best skill and judgment, at all times, in deciding upon the nature of the disease, and the best mode of treatment, and the management generally of the patient.

"The essence of the contract is, that he is to do his best—to yield to the use and service of his patient his best knowledge, skill and judgment, with faithful attention by day and by night as reasonably required. But there are some things that the law does not imply or require. He is not responsible for want of success in his treatment, unless it is proved to result from want of ordinary care or ordinary skill and judgment. He is not a warranter of a cure, unless he makes a special contract to that effect. If he is shown to possess the qualifications stated in the first proposition, to authorize and justify him in offering his services as a physician, then, if he exercises his best skill and judgment, with care and careful observation of the case, he is not responsible for an honest mistake as to the nature of the disease, or as to the best mode of treatment, when there was reasonable ground for doubt or uncertainty.

"If the case is such that no physician of ordinary knowledge or skill could doubt or hesitate, and but one course of treatment would by such professional man be suggested, then any other course of treatment might be evidence of a want of ordinary knowledge or skill, or care and attention, or exercise of his best judgment, and a physician might be held liable, however high his former reputation. If there are distinct and different schools of practice, as Allopathic or Old School, Homœopathic, Thomsonian, Hydropathic or Water Cure, and a physician of one of those schools is called in, his treatment is to be tested by the general doctrines of his school, and not by those of other schools. It is to be presumed that both parties so understand it. The jury are not to judge by determining which school, in their own view, is best. Apply these rules to the evidence.

"Then, as to medical and surgical treatment of the case—was there, or was there not a want of ordinary skill and judgment, such as to render the plaintiff liable within the above rules—such evidence as satisfies you that he either did not possess the education, judgment and skill, which authorized him to undertake the case and enabled him to treat it with ordinary skill, or that he was guilty of that neglect or carelessness in the treatment or investigation of the case which showed that he did not faithfully and honestly apply his skill and knowledge, and best judgment."

Defendant requested the court to give the following instruction:

"A physician who, upon request and in consideration of being paid for his services, takes charge of the case of a diseased person, warrants that he possesses and promises to exercise the knowledge, skill and care requisite to enable him to understand the nature of of his disease, and to treat it properly, but the degree of such knowledge, skill and care, is not that which is possessed and exercised by physicians of the highest knowledge, skill and care, but it is that possessed by physicians of ordinary knowledge, skill and care."

The judge declined to give this, except as given in former instructions.

The judge in his charge also instructed the jury, that in cases where authorities differ, or "doctors disagree," the competent physician is only bound to exercise his best judgment in determining which course is, on the whole, best.

Verdict for plaintiff for the amount of his bill, to which rulings and refusals the defendant excepted.

The case on the exceptions was argued before the Law Court, at the May term, 1862, and the rulings of the judge at the trial were sustained.

The opinion of the court was drawn up by Appleton, C. J.

"The instructions given were correct. A plaintiff in a suit against a physician for malpractice, must prove that the defendant assumed the character and undertook to act as a physician, without the education, knowledge and skill, which entitled him to act in that capacity; that is, he must show that he had not reasonable or ordinary skill; or, he is bound to prove, in the same way, that having such knowledge and skill, he neglected to apply them with such care and diligence, as, in his judgment, properly exercised, they must have appeared to require; in other words, that he neglected the proper treatment from inattention and carelessness. *Leighton* v. *Sargent*, 7 *Foster*, 460. The same facts which would authorize a recovery for malpractice would constitute a defense in a suit for professional services. Physicians do not warrant the success of their prescriptions. 'The law,' remarks Mr. Justice WOODWARD, in *McCandless* v. *McWha*, 22 *Penn.* 261, 'demands *qualifications* in the profession practical; not extraordinary skill, such as belongs only to few men of rare genius and endowments, but the degree which ordinarily characterizes the profession.' The same views of the law were laid down in *Simonds* v. *Heny*, 39 *Maine*, 155.

"The instructions given were in accordance with the settled principles of law. The one requested had been given in substance. If other instructions had been desired, they should have been requested." *Exceptions overruled.*

RICE, CULLING, DAVIS, and KENT, J. J., concurred.

*Wilmot* v. *Howard*, 39 *Vermont*, 447.

This was an action on the case against the defendant for damages occasioned to the plaintiff by want of skill of the defendant as a surgeon, in setting the plaintiff's arm, and for negligence and inattention to the same after it was set.

Plea, the general issue, and trial by jury, June term, 1866.

The plaintiff is the minor son of the *next friend*, Daniel C. Wilmot, and was, at the time of the injury complained of, about seven years of age, and resided with his father.

The counsel for the plaintiff introduced evidence tending to show, that about the 9th of June, 1863, the plaintiff fell and broke one of the bones of the forearm, and that the father of the boy took him to the defendant's, to have his arm set, and otherwise dressed and attended to; that the defendant, who professed to be a surgeon, undertook to set and take charge of the fractured arm; that he did set it, but that in doing so, and in dressing the arm, he did not use ordinary skill, and that by reason of an improper bandage, and putting on the bandage too tight, it caused pain and suffering to the plaintiff, and mortification, decay, and the entire loss of the use of the arm. There was evidence tending to show, that when the defendant set the arm he dressed it by first winding a bandage around the arm very tight, from near the hand to very near or quite to the elbow, then put on the splints outside of this, then another bandage wound around the arm tight from near the hand to the elbow, over the splints; and the testimony of several practicing surgeons was given, which tended to show that an inner bandage was improper, unnecessary, and detrimental, and that the tight manner in which this was put and kept on, in their opinion, caused the arm to perish and mortify. The plaintiff's counsel further gave evidence tending to prove, that as the father was about to leave the office of defendant, it was, at the defendant's suggestion, agreed and arranged by them—this being Wednesday—that on the succeeding Sunday, the father of the boy should take him to the office of the defendant to have the arm dressed or whatever else its condition should require; that immediately after the arm was set, it became and continued very painful, on account of the improper and unskillful bandage and dressing; that on Friday following, the defendant was passing by the house of the father of the boy, when the father called the defendant in to see the arm; and that he then informed him that the bandage was too tight, and that it caused great pain to the arm, and that it had done so ever since he set it; that the boy had been in great distress and complained of the bandage being too tight, and of great pain in his arm, and called the defendant's attention to the fact that the hand of the broken arm was swollen and blistered on the fingers. The evidence tended to show that the hand had become purple, and that the defendant said he would not undo the arm; that he then called his attention to one of the splints being up so high as to interfere with the arm at the elbow, and that the defendant thereupon unbound the outer bandage far enough to slip down the inner splint so that it would not prevent bending the elbow, and put on the bandage again as it was before, after thus adjusting the splint on the inside of the arm.

The plaintiff further gave testimony tending to prove that the defendant then told the plaintiff he need not fetch the boy to his office at all, and agreed to call and see the arm the then next Monday or Tuesday at said Wilmot's; that on the following Tuesday the defendant passed by the house of the plaintiff and did not call, and never after that called upon or saw the arm.

The defendant gave testimony tending to prove that on the Friday when the father of the plaintiff called defendant in to see the arm, it was agreed that the father of the plaintiff, on the succeeding Sunday, should take the plaintiff to the defendant's office to have his arm dressed if it needed dressing, and that the defendant did not agree to call at the house of the father of the plaintiff.

And the defendant further introduced testimony tending to prove, that on several occasions, one of which was on the next Sunday succeeding said Friday, the father of the plaintiff, not in the presence of the defendant, told individuals that he had agreed to take the plaintiff to the defendant's office on the next Sunday after the said Friday to have the arm examined and dressed, if it needed dressing.

Wilmot testified that he never made any such statement, but admitted that he had stated, on one or two occasions, that at the time the defendant first set the arm, it was understood he was to take the boy to the defendant's office the next Saturday or Sunday after it was first set by the defendant. It was admitted on the part of the plaintiff, that the plaintiff was not taken to the defendant's office, as the defendant claimed the agreement was, and that the defendant was not called upon or requested, after said Friday to attend upon the plaintiff, and that the defendant never did see or attend upon the arm after that time.

The testimony on the part of the plaintiff tended to prove, that the arm was not well and properly dressed, and that the plaintiff has lost the use of his arm by reason of the want of proper skill in setting the same, on the part of the defendant, and by reason of negligence and inattention to the same, on the defendant's part after it was set.

The defendant's testimony tended to prove that the plaintiff's arm was well and skillfully dressed and set, and that the loss of the use of the arm resulted from the peculiar injury of the same, at the time it was broken, and from the fault and negligence of the father of the plaintiff in not bringing the plaintiff to the office to be dressed and attended to as he had agreed to do, and from other want of proper care and attention.

There was other evidence as to what was said and done and agreed upon when the defendant came into the father's house on Friday to see the arm, upon the part of both parties; also as to the effect of the defendant's treatment, the injury to the arm on account of the manner of dressing, negligence, etc., of the defendant, and the treatment by other surgeons.

The counsel for the defendant claimed to the jury in argument that if the damage to the arm resulted in whole or in part from the mismanagement and negligence of those having the care and management of the plaintiff, that the plaintiff is not entitled to recover; and stated to the jury that he presumed the court would so charge. The court treated this as properly raising and presenting the question. The court did not so charge.

No other requests were made by the defendant's counsel.

The court charged the jury fully upon all points presented by the evidence, and no exceptions were taken to any neglect or omission to charge, or to the charges as given, except as heretofore stated. In the course of the charge the court told the jury that there was an implied obligation on a man who holds himself out to a community as a surgeon, and practicing that profession, that he should possess the proper skill in surgery, that is, not the highest degree of skill that by study and experience the profession is susceptible of, or that is possessed by the most eminent surgeons, but the ordinary skill of the profession generally, such degree of knowledge and skill as surgeons commonly possess, such as is common among surgeons who practice that profession; but that want of such skill would not make the surgeon liable, unless it was also shown that the injury complained of resulted from and was caused by the want of such skill as a surgeon is required to possess.

The defendant's counsel excepted to this charge, so far as it relates to the degree of knowledge and skill a surgeon should possess.

As to the declarations of Daniel C. Wilmot, which the defendant claimed to have proved, to the effect that he, Wilmot, had agreed on that Friday to take the boy to the defendant's office the next Sunday thereafter, no request was made to charge in relation to the evidence as to such declarations, but both of the counsel who argued the case for the defendant, stated to the jury that they did not claim that such declarations were evidence in chief to prove the fact of such agreement, but that they were evidence tending to discredit and impeach Daniel C. Wilmot, and the case was so argued to the jury on both sides, *and the court so charged the jury.* After the court had charged the jury, the defendant's counsel excepted to the charge on this point, claiming then that such subsequent declarations of Daniel C. Wilmot were evidence in chief. The court then informed defendant's counsel that the charge was as they claimed in their argument to the jury, but that the court would state in the exceptions the facts, so that the Supreme Court might decide whether under such circumstances they were entitled to have their exceptions allowed, and if so, the error, if any, might be corrected by the Supreme Court. The facts were stated for that purpose.

As to what the defendant's counsel claimed, as heretofore stated, as to the effect of mismanagement, negligence, or want of proper care and attention on the part of the plaintiff, and those, other than the defendant, having the care and management of him, the court charged the jury, as to this branch of the case, and its effect on the rights of the parties and upon the damages to the satisfaction of the defendant, and so that no exception was taken, except the court did not charge that if damage or injury resulted, *in part*, from the mismanagement and negligence of those having the care and management of the plaintiff, that the plaintiff could not recover, — and for the omission so to charge, the defendant excepted.

The jury returned a verdict for the plaintiff. *Appealed.*

For the defendant :—The negligence of those having the care, charge and custody of the plaintiff was not merely *permissive*, like permitting, negligently, a child to play in the public highway, or otherwise be in the way of danger; but is active, affirmative negligence.

In a case of mere permissive negligence,—permitting a child to be improperly in the highway,—it was held in *Robinson* v. *Cone*, 22 *Vt.* 213, that if the child were injured by the negligence of the defendant, he would not be precluded from his redress; that if the defendant knew the child was in the highway, he was bound to exercise proportionate watchfulness and the utmost care. That case was decided mainly upon the authority of the case of *Lynch* v. *Nurdin*, 1 *Ad. & El., N. S.* 28 (41 *E. C. L.* 422), in which a similar decision had been made. And the similar decision in *Birge* v. *Gardiner*, 19 *Conn.* 507, was also made upon the authority of *Lynch* v. *Nurdin;* and upon the authority of these two cases, and upon a like state of facts, the same decision has since been made in Connecticut in the case of *Daley* v. *Norwich & Worcester R. R. Co.*, 26 *Conn.* 91. But the case of *Robinson* v. *Cone* is opposed to the decision in *Hatfield* v. *Roper*, 21 *Wend.* 614, and to the decision in *Wright* v. *Malden & Melrose R. R. Co.*, 4 *Allen*, 283, in both of which, negligence on the part of the parents in allowing the child to be in the way of danger, was held to preclude the recovery, by the child, of damages for injury received.

And the case of *Lynch* v. *Nurdin* has been questioned in *Lygo* v. *Newbold*, 9 *Exch.* 502; *S. C.*, 14 *Eng. L. & Eq.* 507.

But the negligence on the part of the parents in the case at bar is, in its character and consequences, entirely different from the permissive negligence of parents who allow their child to be in the way of danger; and therefore the cases cited above do not control the decisions in this case. Here the parents knew the danger of the child and had the means of preventing the injury, and neglected to use them, and permitted the child to remain in danger, and were also, as the testimony tended to prove, guilty of positive mismanagement in the care of the child.

The case, in its facts, is like the case of *Holley* v. *Boston Gas Light Co.*, 8 *Gray*, 123.

The distinction between the two classes of cases is the difference between permitting a child to be in the way of danger, and permitting a child, known to be in danger, to remain in danger.

And there is this further difference between the case of *Robinson* v. *Cone* and the case at bar: in *Robinson* v. *Cone* the injury to the plaintiff was the result of direct force, exercised and controlled by the defendant; and the reasoning of the court was directed to and upon that state of facts; while, in this case, the negligence on the part of the defendant, if any, consisted in the omission of any affirmative act, and the child was all the time in the care and under the control of its parents.

For the plaintiff:—1. The objection of the defendant that the sayings of the *prochein ami* ought to have been received and considered as testimony in chief, is not well taken. *Vaughn* v. *Porter*, 16 *Vt.* 266.

2. The charge of the court as to the degree of knowledge and skill in his profession a surgeon is required to possess, was correct. *Patten* v. *Wiggin*, *Am. Law Reg.*, 1862–3, 401; *Hilliard on Torts*, 253.

3. The court did not err in omitting to charge the jury that, "if the injuries resulted in whole or in part from the mismanagement of those having the care and management of the plaintiff, the plaintiff could not recover."

1. Even if that be the law applicable to cases of this sort, the defendant was not, under the circumstances of this case, entitled to that charge.

The defense set up was: 1st. That the arm was well and skillfully set and dressed. 2nd. That the loss of the use of the arm resulted from the peculiar nature of the injury, and from the fault and negligence of the father in not bringing the plaintiff to the defendant's office, to be dressed and attended as he agreed to do, and from other want of proper care and attention.

What evidence was given tending to show want of proper care and attention other than that tending to show that the plaintiff was to be taken to the defendant's office to be treated, which was neglected, the exceptions do not state, but we are to conclude that whatever might have fallen out of that character was disposed of in the course of the charge in connection with the whole evidence to the satisfaction of the defendant, so that the court below considered it of no importance to state. We are to presume that the charge was correct, and sufficiently comprehensive, and that what evidence there might have been tending to show want of care, was properly disposed of by specific directions, as was the evidence in relation to the plaintiff being carried to the defendant's office.

2. The exception is not properly before the court. There was no request made that the court would charge in a particular way, and so no refusal. The exception is only to an *omission* to charge. It is not sufficient that the counsel shall say in the course of argument to the jury, that he presumes the court will charge in a particular manner.

3. But the proposition is not sound law, to the extent claimed, and so the court was not bound to notice it. *Vaughn* v. *Porter*, 16 *Vt.* 266.

The opinion of the court was delivered by BARRET, J.

"In this action, the plaintiff claims to recover damages for injury sustained by reason of the unskillful and negligent manner in which the defendant dressed, treated and attended the plaintiff's fractured arm.

"A question is made as to the instructions given to the jury touching certain sayings of the plaintiff's father. Those sayings had been treated, by counsel on both sides, in the argument to the jury, as bearing only on the credit of said father as a witness testifying for the plaintiff to material facts in the case. The court, in the charge, had given instructions conformable to the views taken by counsel in the argument. No prior request had been made to the court on this subject. After the charge had been given, the defendant's counsel requested the court to instruct the jury that said sayings of the father were to be regarded and considered as evidence in chief. These facts are certified to this court for the purpose of having us decide whether the defendant was entitled to exception on this point. In the opinion of this court, the defendant was not entitled to exception. If the defendant claimed any special force, or character, or application for this evidence, he should have made it known before the close of the argument. On the general subject we adhere to the rule pronounced in *Vaughn* v. *Porter*, 16 *Vt.* 266, and re-asserted in *Cady* v. *Owen*, by POLAND, Ch. J., 34 *Vt.* 598. But the present case goes beyond those cited, and asks the court, in favor of the party, to repudiate the character and application which he has claimed for the evidence in his argument to the jury, upon which character and application the counsel for the other side, concurring with his opponent, has also argued the case to the jury, and requires the court to put the evidence to the jury in a new character, in a new application, and to lead to entirely different results from that claimed for it in the argument. This certainly is a novelty in practice. It is understood to be the object of an argument, among other things, to apprise the court of the views and reasons of counsel as to the evidence, as it stands related to the legal propositions involved in the case, and to apprise the opposing counsel of the same things and enable them to present their views and reasons to meet those of the other side; and still further, is it an important and leading object of the argument to bring to the consideration of the jury the various elements and features of the evidence as bearing upon the various propositions of fact which the jury are to pass upon, and aid them in arriving at just results from the evidence as to those propositions.

"The course proposed and pursued by the defendant's counsel in this case, would thwart all these purposes and objects; and not only so, but would directly tend to embarrass the court, mislead opposing counsel, and confuse and confound the jury.

"We, therefore, put our decision of this point solely on the ground that the defendant was not entitled to the exception, without considering whether the view embodied in his unreasonable request was correct or not. We think the court did not err in disregarding the request.

"The point made in the exceptions upon the part of the charge that related to the skill required of a person holding himself out and undertaking to practice as a surgeon, we have no occasion to take time with, as it is not really insisted on and urged in this court. We remark, however, that, taken in its relations to the declaration and the evidence, it would seem to be entirely proper, and as favorable as counsel could claim for their client, unless they would have him take refuge in the character of a quack, from the consequences of his practice as a *professed* surgeon.

"The most important feature of the case is presented by the exceptions to the omission of the court to charge, 'that if the damage or injury to the plaintiff's arm resulted *in part* from the mismanagement and negligence of those having the care and management of the plaintiff, that the plaintiff could not recover.'

"The court had given a full and satisfactory charge upon every other feature and theory of the defense, and of course had told the jury that if the defendant had exercised the requisite skill, care and attention in dressing and treating, and attending to the fracture, he would not be liable; and, also, that if the damage or injury resulted wholly from the fault of those in charge of the plaintiff, the defendant would not be liable. Upon the case as situated under these points and features of the charge, the request not complied with assumes, and was made upon the assumption, that the jury should find that the damage and injury was caused, *in part*, by unskillfulness and negligence of the defendant; and upon the assumption that the defendant would be liable, unless the putting of that point to the jury in the terms of the request would shield him. Every other theory and ground of defense was made available to him by the charge given, and he was found liable notwithstanding. The point, therefore, is this, whether, if the failure of the plaintiff to get a sound arm resulted, *in any part*, from the mismanagement and negligence of those having charge of him, the defendant would not be liable at all in this action.

"This question is to be considered and determined with reference to both the law and the evidence applicable to the point.

"It is to be noticed that upon the evidence, there is no ground of pretense that any mismanagement or negligence had occurred prior to the Friday after the original dressing, at which time the defendant was called in, and examined the condition of the limb and of the patient.

"The evidence, and the respective claims of the parties, as to the argument on that Friday about the plaintiff being seen by the defendant on the following Sunday, or Monday, or Tuesday, were submitted to the jury with satisfactory instructions.

"Hence, the question really is, whether, upon the evidence, the defendant could be found liable in this action, even though the failure of the plaintiff to get a sound arm resulted, *in part*, from the mismanagement or negligence of those having charge of the plaintiff. If he could, then he was not entitled to have his request granted; if he could not, then it should have been granted.

"It seems to us quite clear, that, upon the evidence, the defendant might well have been held liable, even though the jury should have found that the damage to the arm resulted in part from the alleged mismanagement or negligence of those having charge of him.

"If the jury should have found, as they might on the evidence, that the improper manner in which the arm was dressed and kept till the Sunday after the accident, had brought it into such condition that the plaintiff must inevitably have a defective arm, the defendant would be liable to an action, even though it should be found that mismanagement or negligence in those having charge of the plaintiff may have aggravated the case, and rendered the ultimate condition of the arm worse than it otherwise would have been. The cause of action would have become perfected before the alleged mismanagement or negligence would have supervened. There is no pretense that the parents or attendants of the plaintiff had anything to do with the dressing of the arm. If the jury had found that that dressing was such, when continued according to the directions of the defendant, that it would produce a defective arm, and had that effect, then the right of action would have been perfected though the ultimate result might have been aggravated by mismanagement or negligence. In the cases supposed, such supervening mismanagement or negligence would bear only on the measure and amount of damage—not on the right of action.

"If the defendant would have been liable in either of the supposed cases, then of course he was not entitled to have his request granted. In this respect the case would stand by analogy upon the same ground as a common class of cases, particularly for recovery of damages caused by alleged defects in highways. The liability of the town is established, the injury proved, and resulting effects become the subject of inquiry; whereupon the town claims, and endeavors to prove that, owing to mismanagement or negligence in treating the injured party, the consequences have been aggravated. Such showing on the part of the town does not touch the cause and right of action, but only the measure and amount of damages.

"And here it may be well to remark that this just illustrates and makes plain the distinction to be taken between the case before us, upon the precise point made by the exceptions, and all the cases cited by the defendant's counsel as applicable to it.

"In those cases the alleged negligence on the part of the plaintiff was simultaneous and co-operating with the alleged fault of the defendant, an element in the very transaction which constitutes the alleged cause of action. The contributory negligence on the part of the plaintiff, in all the cases, that has been held to preclude his right of recovery, has entered *the creation of the cause* of action, and not merely supervened upon it, by way of aggravating the damaging results.

"These views leave this case to stand upon common principles by which a person is subjected to liability for the consequences of his wrongful acts and neglect, and as the case is made up it would seem that the defendant has had accorded to him every legitimate ground and means of defense. The exceptions state, as to what the defendant's counsel claim as to the effect of mismanagement, negligence or want of proper care and attention on the part of the plaintiff, and those other than the defendant having the care and management of him, 'the court charged the jury, as to this branch of the case, and its effect on the rights of the parties and upon the damages, to the satisfaction of the defendant, and so that no exception was taken except the court did not charge that if the damage or injury resulted, in part, from the mismanagement and negligence of those having the care and management of the plaintiff, the plaintiff could not recover.'

"In the view we take of the case we do not find occasion to go into any general discussion of the subject as it is involved in, or related to, the cases that have been cited. This case stands upon simple and familiar principles in no respect in conflict with any of the decided cases, and directly sustained by some, so far as they stand upon concurring analysis. *Robinson* v. *Cone*, 22 *Vt.* 213; *Birge* v. *Gardiner*, 19 *Conn.* 507; *Daley* v. *Norwich R. R. Co.*, 26 *Conn.* 91."

The "degrees" of negligence are ably discussed in the case of the *Steamboat New World* v. *Frederick G. King*, 16 *Howard's* (*U. S. S. C.*) *R.* 469, where, also, will be found a large number of cases cited, bearing upon the subject. See for same case, *Livingston's Law Mag.*, *March*, 1855, *p.* 163.

The following case is from the English Courts, and with the exception of the case of *Slater* v. *Baker* is, I believe, the oldest on record.

*Seare* v. *Prentice*, 8 *East*, 448, April 29, 1807, 47 *G.* 3.

This was an action on the case brought by the plaintiff, a shoemaker, against the defendant, whom he had employed as a surgeon, for negligently, ignorantly and unskillfully reducing a dislocated elbow and fractured arm of the plaintiff, of which he had undertaken the cure. The case was tried before HEATH, J., at the last assizes at Hertford; and a verdict having been given for the defendant, under the direction of the learned judge, that direction was now impeached and a rule *nisi* for setting aside the verdict and granting a new trial was moved for by Gurney, upon the ground that there was evidence laid before the jury of the unskillful treatment of the plaintiff by the defendant; but that they were told by the learned judge, that unless *negligence* were proved, they could not examine into the *want of skill*; and the evidence he now admitted, did not substantiate the charge of *negligence* though it proved the want of skill. And he referred to *Slater* v. *Baker*, 2 *Wilson*, 359, to show that an action lay against a surgeon for ignorance and unskillfulness in his profession, and to *Bull, N. P.* 73, where the general rule is laid down, that in all cases where damages accrue to another by the negligence, ignorance or misbehavior of a person in the duty of his trade or calling, an action on the case will lie; as if a farrier kill my horse by bad medicine, or refuse to shoe, or prick him in the shoeing.

The court granted a rule *nisi.* And now upon the judge's report being read, the case appeared to be this:

The plaintiff's brother-in-law proved on his behalf, that on the 2nd of April, 1805, the defendant attended the plaintiff, who had fallen from a horse, and told the defendant that his arm was broken; the defendant said he thought the arm, which was swollen, was not broken, and applied vinegar to it and bound it with tape. That the plaintiff was under the defendant's care for ten weeks without being cured; he then applied to Mr. Kingston, another surgeon, and after sometime could work, and put his arm to his head. On cross-examination the same witness proved that the defendant was first sent for at night and came directly; that he regularly attended the plaintiff every day but one, till the latter applied to Mr. Pidcock, another surgeon, who about nine or ten days after the accident, attended and assisted with the defendant in setting the elbow.

Mr. Kingston, the surgeon, then proved that in July, 1805, the plaintiff was brought to him, a cripple in his arm, one bone of which was broken obliquely below the elbow; that the plaintiff's arm was almost straight; he could not turn his wrist, and had no motion in the elbow; that the witness broke the callus and set it again, and made (what the witness himself described as) a very fine cure, which was spoken of about the country. He imputed the failure of the defendant in his attempt to cure the plaintiff to *negligence and carelessness;* an apprentice boy, (he said) might have known better; that the bone might have been set within five hours after the accident; though he admitted that the swelling, if much, must first be reduced, which might take a fortnight. And he recommended the plaintiff to bring an action. He also spoke of a conversation with the defendant, who considered it as a very difficult dislocation to reduce; and said he would make a compensation to the plaintiff.

The learned judge told the jury that the gist of the action was negligence, of which direct evidence might be given; or it might be inferred by the jury, if the defendant had proceeded without any regard to the common ordinary rules of his profession. *That unskillfulness alone, without negligence, would not maintain the action.* And that he was at a loss to state to the jury what degree of skill ought to be required of a village surgeon. But that, whether or not his directions were accurate in this respect, at any rate the witness Kingston imputed only *negligence* and *carelessness* to the defendant and Pidcock in not discovering the fracture of the bone of the arm when they reduced the dislocated elbow; which there was no doubt was properly reduced; and that considering all the circumstances of the case, he did not think that such gross negligence was imputable to the defendant as to make him liable in damages to the plaintiff. The report concluded by stating that the jury found a verdict for the defendant, much to the judge's satisfaction; who intimated that the vaunting language of the witness Kingston must have diminished his credit with the jury.

Shepherd, Sergt., and Espinasse, were now to have shown cause; but though all the court seemed to be satisfied, as well now as when the rule was moved for, that the action well lay for unskillfulness in the profession of a surgeon; yet upon a revision of the evidence as reported, they asked of the plaintiff's counsel what evidence there was of *want of skill* in the defendant: Kingston the surgeon only imputing to him *negligence* and *carelessness* which the learned judge had stated to be a ground of action, and had left to the jury for their consideration; but which the jury had negatived; as indeed the evidence well warranted them in doing.

Gurney, in support of the rule, said that it was to be collected from the whole of Kingston's evidence that he imputed want of skill to the defendant; and that was shown by the expression used by him, that an apprentice boy might have known better. That so much skill at least was required of a surgeon as to be able to tell whether or not an arm was broken or an elbow dislocated. But it was enough that the question of want of skill was wholly withdrawn from the consideration of the jury.

Lord Ellenborough, C. J. "The surgeon who was examined specifically imputed failure of cure to *negligence* and *carelessness*, whatever other expressions he may have used in the manner of giving his evidence, upon which the learned judge has commented. Therefore, however we may differ from the learned judge, as I certainly do, in thinking that an ordinary degree of skill is necessary for a surgeon who undertakes to perform surgical operations, which is proved by the case of *Wilson*, and indeed all analogous authorities, in the same manner as it is necessary for every other man to have it in the course of his employment; as a farrier who undertakes to cure my horse must have common skill at least in his business, and that is implied in his undertaking; and although I am ready to admit that a surgeon would be liable for *crassa ignorantia*, and would be justly responsible in damages for having rashly adventured upon the exercise of a profession without the ordinary qualification of skill, to the injury of a patient; yet the question did not arise upon the evidence in this case; for no want of skill was imputed to the defendant. and therefore the opinion of the learned judge upon that point does not affect the merits of the verdict upon the evidence in the cause."

The other judge concurred, and GROSE, J., referred to 3 Black. Com. (ch. 9, pp. 163–4,) as confirming the general doctrine.*

(To be continued.)

* Vide Esp. Dig. 601, or vol. 2, p. 222, of New York edition, *Lipscombe* v. *Holmes*, 2 *Campb.* 441, and reporter's note thereto, pp. 442–3.

